# A Systematic Review and Meta-Analysis of Independent Predictors for Acute Respiratory Distress Syndrome in Patients Presenting With Sepsis

**DOI:** 10.7759/cureus.37055

**Published:** 2023-04-03

**Authors:** Abshiro H Mayow, Fatima Ahmad, Muhammad Sohaib Afzal, Muhammad Usama Khokhar, Daneyal Rafique, Sai Krishna Vallamchetla, Sujith K Palleti, Faraz Saleem

**Affiliations:** 1 Medicine, St. George's University School of Medicine, St. George's, GRD; 2 Medicine, Washington University of Health and Science, Washington, DC, USA; 3 Medicine, Louisiana State University Health Sciences Center, Shreveport, USA; 4 Internal Medicine, King Edward Medical University, Lahore, PAK; 5 Internal Medicine, Mayo Hospital, Lahore, PAK; 6 Internal Medicine, Army Medical College, National University of Medical Sciences (NUMS), Rawalpindi, PAK; 7 Medical School, All India Institute of Medical Sciences, Bhopal, Bhopal, IND; 8 Nephrology, Edward Hines Jr. Veterans Administration Hospital, Hines, USA; 9 Nephrology, Loyola University Medical Center, Maywood, USA; 10 Internal Medicine, California Institute of Behavioral Neurosciences & Psychology, California, USA; 11 Internal Medicine, Akhtar Saeed Medical and Dental College, Lahore, PAK

**Keywords:** hospitalization, systematic review and meta-analysis, sepsis, acute respiratory distress syndrome, predictors

## Abstract

The current meta-analysis was conducted to determine the predictors of acute respiratory distress syndrome (ARDS) in patients with sepsis. The present meta-analysis was conducted in accordance with the MOOSE (Meta-analysis of Observational Studies in Epidemiology) guidelines. We conducted a systematic search using the PubMed, Cochrane Library, and EMBASE databases for studies published between 1 January 2000 and 28 February 2023 that assessed the predictors of ARDS in patients with sepsis. We used key terms such as "predictors," "acute respiratory distress syndrome," and "sepsis" to search for relevant articles. Our search was limited to human studies published in English. A total of six studies were included in this meta-analysis. Of the six studies, four were retrospective and two were prospective. The pooled incidence of ARDS was 11.27%. We identified six factors with a consistent and statistically significant association with ARDS, including sequential organ failure assessment (SOFA) score, Acute Physiology and Chronic Health Evaluation (APACHE) II score, pulmonary sepsis, smoking, pancreatitis, and C-reactive protein. Age, diabetes, and chronic obstructive pulmonary disease (COPD) were not found to be significantly associated with ARDS in this patient population. It is important for healthcare providers to consider these predictors when assessing patients with sepsis and septic shock to identify those at high risk for developing ARDS and implement appropriate preventive measures.

## Introduction and background

Around the world, approximately 19 million individuals are affected by sepsis annually, a serious medical condition that occurs when the immune system responds abnormally to an infection, resulting in organ dysfunction and potentially life-threatening consequences [1]. Acute respiratory distress syndrome (ARDS) is one of the devastating complications of severe sepsis and is associated with a high case-fatality rate [2]. It occurs due to the systemic infection that produces general inflammation and releases inflammatory mediators that lead to severe lung injury [3]. There are two primary pathophysiologic changes associated with ARDS, which occur to varying degrees. One is the accumulation of protein-rich fluid in the alveolar space caused by damage to the local alveolar epithelium. The other is the leakage of fluid into the pulmonary interstitium through the capillary endothelium caused by systemic inflammation. Direct ARDS typically causes more damage to the alveolar epithelium and less damage to the capillary endothelium than indirect ARDS [4,5]. Like sepsis, ARDS is associated with a high mortality rate of 30 to 40%, and patients with sepsis-induced ARDS have a higher death rate than those with other risk factors [6]. Survivors of sepsis-induced ARDS can experience physiological or functional sequelae, such as impaired cognition, difficulty concentrating, and impaired memory [7].

It has been reported that patients who have sepsis-induced ARDS have a more unfavorable prognosis compared to those who do not have ARDS [8] because there are currently no effective treatments available for the condition [9]. Due to the rapid progression of these clinical conditions, healthcare providers have shifted their management strategy to concentrate on preventing the occurrence of ARDS [10]. One important way to prevent the development of ARDS in sepsis patients is to identify the high-risk population at an early stage and initiate treatment promptly.

Although several risk factors have been identified through previous studies, there is still a lack of consensus on the predictors of ARDS in sepsis patients, and the sample size of these studies was small. The present meta-analysis will be useful to achieve greater statistical power and generate more robust conclusions. Therefore, conducting a meta-analysis to identify the predictors of ARDS in sepsis patients could provide valuable insights into risk assessment and help guide clinical decision-making. Moreover, such findings could inform the development of targeted interventions to reduce the incidence of ARDS in this population and improve patient outcomes. Therefore, the present meta-analysis was conducted to determine the predictors of ARDS in patients with sepsis.

## Review

Methodology

The present meta-analysis was conducted in accordance with the MOOSE (Meta-analysis of Observational Studies in Epidemiology) guidelines.

Study Selection

We conducted a systematic search using the PubMed, Cochrane Library, and EMBASE databases for studies published between 1 January 2000 and 28 February 2023 that assessed the predictors of ARDS in patients with sepsis. We used key terms such as "predictors," "acute respiratory distress syndrome," and "sepsis" to search for relevant articles. Additionally, we searched the reference lists of the included studies. Two authors conducted the search independently, and any disagreements were resolved through discussion with the principal investigator.

Selection Criteria

For the present meta-analysis, we included prospective or retrospective cohort studies that included patients with sepsis and assessed predictors of ARDS. We excluded case-control studies, reviews, case reports, and case series. We included studies published in the English language only. We also excluded studies that involved patients with conditions other than sepsis. We conducted an initial screening using abstracts and titles after removing duplicates, followed by a full-text review to identify studies for further review.

Data Extraction and Quality Assessment

Two authors independently extracted data related to the predictors of ARDS in patients diagnosed with sepsis from the included studies for potential meta-analysis. Information extracted included the author's name, year of publication, study design, number of participants, and estimates of associations between ARDS and independent predictors. We used a Microsoft Excel sheet (Microsoft, Washington, United States) to record the outcomes of the included studies. The quality of the studies was independently assessed by two authors independently using the New castle-Ottawa Scale (NCOS) for observational studies. NCOS scale rates studies on three major domains including exposure, comparability, and selection.

Statistical Analysis

We used RevMan Review Manager Version 5.4.1 (The Cochrane Collaboration, The Oxford, United Kingdom) for our analysis. As predictors were reported both as continuous and categorical variables, we analyzed them separately. To determine the association between independent categorical variables and ARDS, we calculated odds ratios (OR) comparing the prevalence of predictors in patients with ARDS versus those without ARDS (diabetes, chronic obstructive pulmonary disease, pulmonary sepsis, smoking status, pancreatitis). We pooled crude ORs using a Mantel-Haenszel random-effects model. For continuous predictors, we calculated the mean difference and 95% confidence interval (CI) between patients with ARDS and those without ARDS (age, C-reactive protein (CRP), acute physiology, and chronic health evaluation (Acute Physiology and Chronic Health Evaluation (APACHE) II score, sequential organ failure assessment (SOFA) score). We considered a p-value <0.05 as significant. We used a random-effects model for analysis due to expected heterogeneity among study results. We assessed heterogeneity using the I-square statistics and Cochran-Q statistics and considered a p-value of <0.1 as significant for heterogeneity.

Results

**Figure 1:**
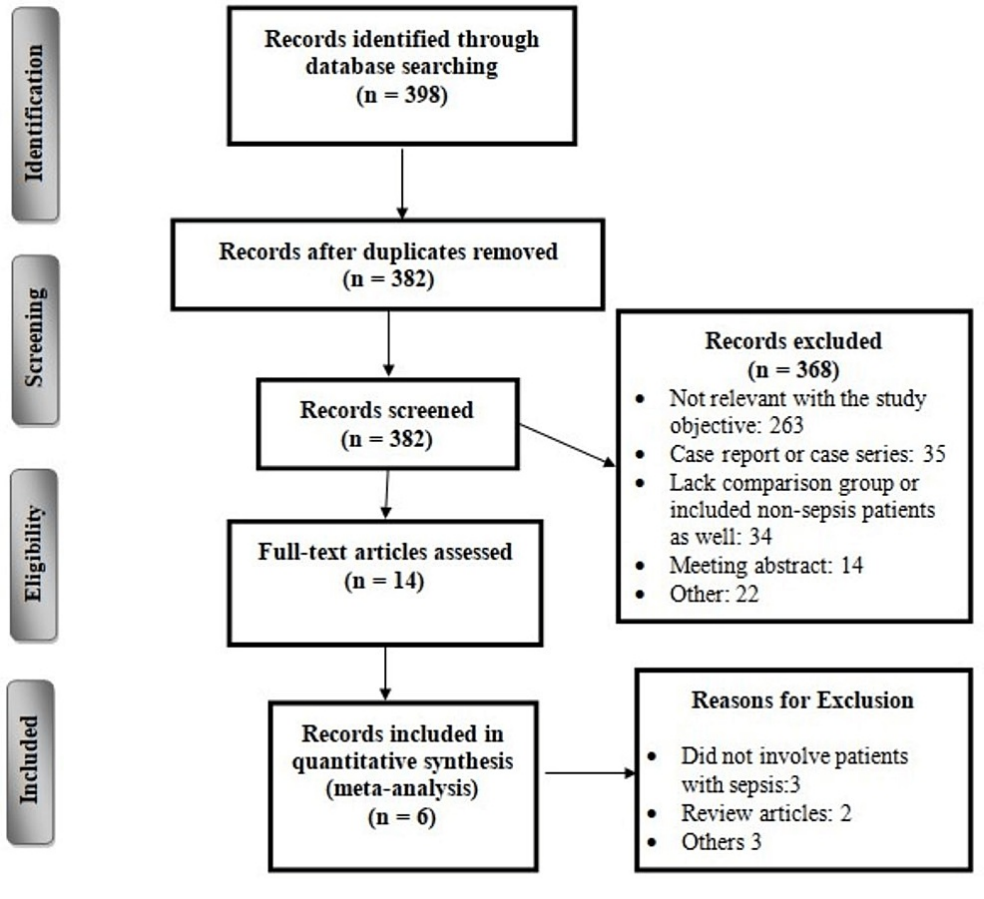
Study selection process

The online database search identified 398 citations. After initial searching based on abstracts and titles, 14 publications remained for full-text assessment. After a close assessment of these studies and application of eligibility criteria, a further eight studies were excluded. The detailed selection process is shown in Figure 1. Of the six studies, four were retrospective and two were prospective. Table 1 shows the characteristics of included studies. Two studies were conducted in China, two in the United States, and one each in Japan and South Korea. The pooled incidence of ARDS was 11.27%. The incidence of ARDS reported in individual studies in the present analysis ranged from 6.16% to 33.84%. Table 2 shows the quality assessment of the included studies.

**Table 1:** Characteristics of included studies ARDS: acute respiratory distress syndrome

Author Name	Year	Region	Study Design	Groups	Sample Size	ARDS (%)
Iriyama et al. [11]	2020	Japan	Retrospective	ARDS	85	14.31
				No-ARDS	509	
Li et al. [12]	2021	China	Prospective	ARDS	41	27.33
				No-ARDS	109	
Mikkelsen et al. [13]	2013	United States	Retrospective	ARDS	48	6.17
				No-ARDS	730	
Nam et al. [14]	2019	South Korea	Retrospective	ARDS	22	17.60
				No-ARDS	103	
Seethala et al. [15]	2017	United States	Prospective	ARDS	156	6.16
				No-ARDS	2378	
Shi et al. [16]	2022	China	Retrospective	ARDS	179	33.84
				No-ARDS	350	

**Table 2:** Quality assessment of included studies

Author Name	Selection	Comparability	Outcome	Overall
Iriyama et al. [11]	3	1	2	Good
Li et al. [12]	3	1	2	Good
Mikkelsen et al. [13]	3	2	2	Good
Nam et al. [14]	2	1	2	Fair
Seethala et al. [15]	3	2	2	Good
Shi et al. [16]	3	2	1	Fair

Predictors of ARDS in patients with sepsis

Age

**Figure 2:**
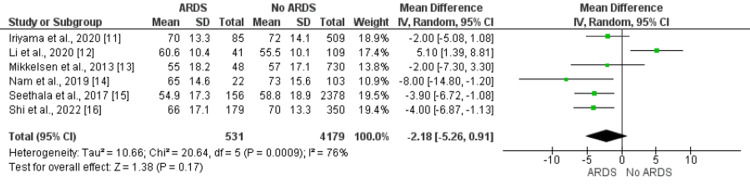
Relationship of age with the development of ARDS. Mean difference > 0 shows higher age in ARDS patients compared to no ARDS patients ARDS: acute respiratory distress syndrome Sources: References [11-16]

Age was not significantly associated with ARDS in patients with sepsis. Overall, a pooled meta-analysis showed that mean age was lower in patients developing ARDS compared to their counterparts as shown in Figure 2. Three of the included studies showed age is significantly lower in patients developing ARDS [14-16], while one study reported significantly higher age in ARDS patients compared to non-ARDS [12]. Two studies did not report any significant difference in age between the two groups [11-13].

APACHE-II Score

**Figure 3:**
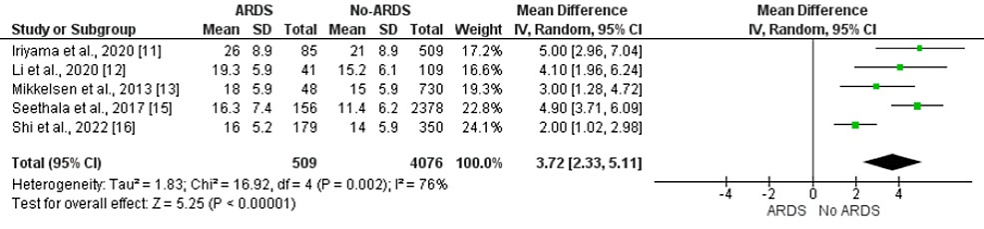
Effect of APACHE II score on the development of ARDS. Mean difference > 0 shows higher APACHE II score in ARDS patients compared to no ARDS patients ARDS: acute respiratory distress syndrome; APACHE II: Acute Physiology and Chronic Health Evaluation II score Sources: References [11-13,15,16]

Five studies compared the APACHE-II score between patients who developed ARDS and patients who did not develop ARDS. The mean APACHE score was significantly higher in patients who developed ARDS compared to non-ARDS patients (MD: 3.72, 95% CI: 2.33, 5.11) as shown in Figure 3. Significant heterogeneity was reported among the study results (I-square: 76%, p-value: 0.002). All five studies compared the APACHE-II score between ARDS and non-ARDS patients and reported significantly higher APACHE-II scores in patients developing ARDS [11-13,15,16].

SOFA Score

**Figure 4:**
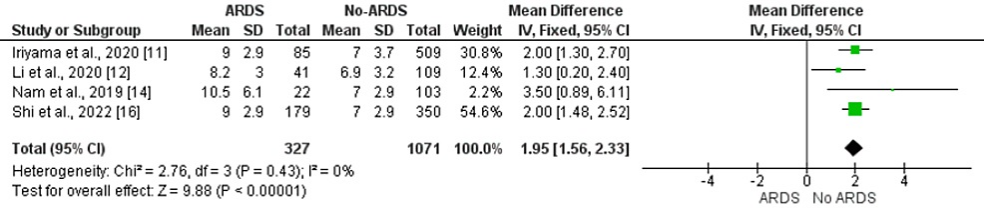
Effect of SOFA score on the development of ARDS. Mean difference > 0 shows higher SOFA score in ARDS patients compared to no ARDS patients ARDS: Acute respiratory distress syndrome; SOFA: sequential organ failure assessment Sources: References [11,12,14,16]

Four studies compared SOFA scores between ARDS patients and non-ARDS patients. The pooled mean SOFA score was significantly higher in patients developing ARDS compared to their counterparts (MD: 1.95, 95% CI: 1.56, 2.33). No significant heterogeneity was reported among the study results (I-square: 0%, p-value: 0.43) as shown in Figure 4. All four studies assessed this independent predictor and found significantly higher SOFA scores in patients developing ARDS [11,12,14,16].

Site of Infection (Pulmonary Sepsis)

**Figure 5:**
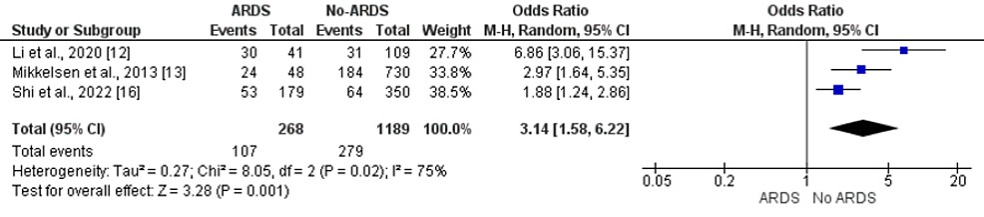
Effect of pulmonary site of infection on the development of ARDS. Odds ratio > 1 shows higher odds of pulmonary sepsis in ARDS patients compared to patients without ARDS ARDS: acute respiratory distress syndrome Sources: References [12,13,16]

Patients developing ARDS had a significantly greater frequency of pulmonary sepsis compared to patients without ARDS (39.92% vs. 23.46%). The odds of pulmonary sepsis are 3.14 times higher in ARDS patients compared to non-ARDS patients (OR: 3.14, 95% CI: 1.58, 6.22). Significant heterogeneity was reported among the study results (I-square: 75%, p-value: 0.02) as shown in Figure 5. All three studies assessed the impact of this predictor and found a higher prevalence of pulmonary sepsis in patients with ARDS compared to non-ARDS [12,13,16].

Diabetes

**Figure 6:**
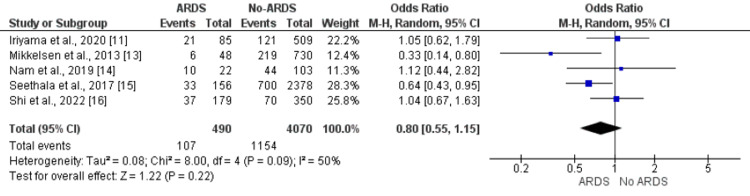
Effect of pulmonary sepsis on the development of ARDS. Odds ratio > 1 shows higher odds of diabetes in ARDS patients compared to patients without ARDS ARDS: acute respiratory distress syndrome Sources: References [11,13-16]

Five studies included in the meta-analysis assessed the impact of diabetes on ARDS in patients with sepsis. No significant difference was reported in the prevalence of diabetes between patients who developed ARDS and patients who did not develop ARDS (OR: 0.80, 95% CI: 0.55, 1.55) as shown in Figure 6. Significant heterogeneity was found among the study results (I-square: 50%, p-value: 0.09).

Chronic Obstructive Pulmonary Disease (COPD)

**Figure 7:**
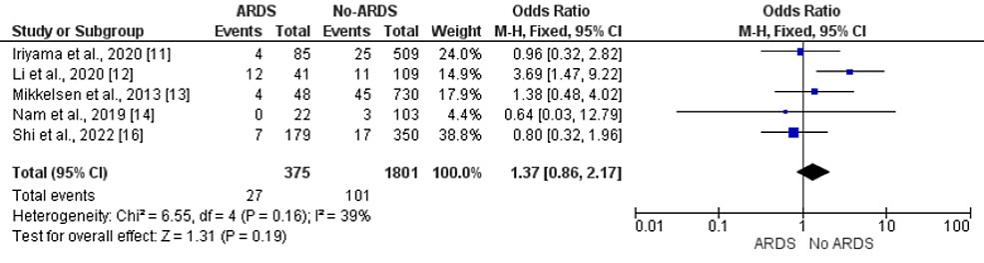
Effect of COPD on the development of ARDS. Odds ratio > 1 shows higher odds of COPD in ARDS patients compared to patients without ARDS ARDS: acute respiratory distress syndrome; COPD: chronic obstructive pulmonary disease Sources: References [11-14,16]

Pooled analysis of five studies showed no significant impact of COPD on ARDS in patients with sepsis (OR: 1.38, 95% CI: 0.72, 2.64) as shown in Figure 7. No significant heterogeneity was found among the study results (I-square: 39%, p-value: 0.16).

Smoking Status

**Figure 8:**
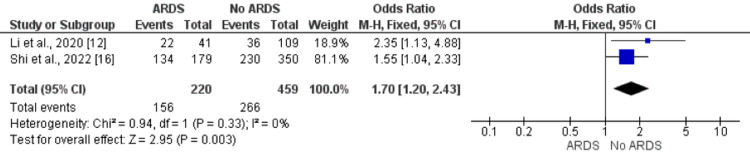
Effect of smoking on the development of ARDS. Odds ratio > 1 shows higher odds of smokers in ARDS patients compared to patients without ARDS ARDS: acute respiratory distress syndrome Sources: References [12,16]

Pooled analysis of five studies showed odds of smokers are 1.70 times greater in patients who developed ARDS compared to patients who did not develop ARDS (OR: 1.70, 95% CI: 1.20, 2.43) as shown in Figure 8. No significant heterogeneity was found among the study results (I-square: 0%, p-value: 0.33).

Other Predictors Independently Associated With ARDS in Patients With Sepsis

**Figure 9:**
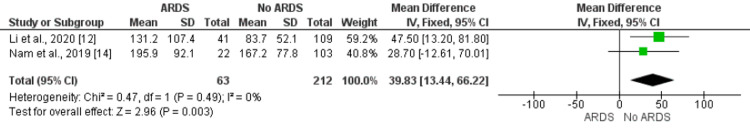
Effect of C-reactive protein on the development of ARDS. Mean difference > 0 shows higher C-reactive protein in ARDS patients compared to no ARDS patients ARDS: acute respiratory distress syndrome Sources: References [12,14]

**Figure 10:**
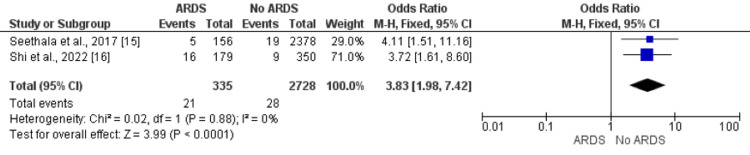
Effect of pancreatitis on the development of ARDS. Odds ratio > 1 shows higher odds of pancreatitis in ARDS patients compared to patients without ARDS ARDS: acute respiratory distress syndrome Sources: References [15,16]

Two studies assessed the relationship between pancreatitis and C-reactive on the development of ARDS in patients with sepsis. A pooled analysis reported a significantly higher mean of CRP in patients with ARDS compared to non-ARDS as shown in Figure 9. Similarly, a higher frequency of pancreatitis was reported in patients with ARDs compared to patients who did not develop ARDS as shown in Figure 10.

Discussion

This is the first study to systematically review the literature on predictors of ARDS in patients with sepsis. We identified six factors with a consistent and statistically significant association with ARDS, including SOFA score, APACHE II score, pulmonary sepsis, smoking, pancreatitis, and CRP. In the present meta-analysis, a pooled incidence of ARDS in sepsis patients reported in the present meta-analysis was 11.27%.

The present meta-analysis found that patients mean age in patients with sepsis-induced ARDS was lower compared to non-ARDS patients. Three of the included studies have reported lower mean age in patients with sepsis-induced ARDS. ARDS is a clinical condition characterized by severe inflammatory responses conveyed by immune activation [17]. A group of structural and functional changes in the immune system, like reduced response to inflammation and vaccination response, are considered to be important components of aging [18]. This reduced response might be one of the important reasons why the age of ARDS patients is lower in the majority of the studies. Future studies need to focus on this clinical phenomenon in critically ill patients.

The present analysis confirmed that the APACHE II and SOFA scores were related to having ARDS consistent with all the results of individual studies included in the present meta-analysis [11-16]. The APACHE II score is a comprehensive tool that evaluates the severity of a patient's illness by taking into account their current vital signs, laboratory values, age, and medical history. This score is useful in assessing patients and determining the appropriate level of diagnostic and therapeutic intervention needed [19]. Several studies have found that the APACHE II score is one of the important prognostic biomarkers of death in critically ill patients [20,21]. Notably, different studies used the APACHE II score within 24 hours of admission, which can be useful in classifying patients at high risk of developing a particular condition [19]. A prospective study also found that APACHE II scores correlated with the severity of the disease and can be a useful indicator of the risk for ARDS in patients with sepsis [12]. Therefore, future studies need to be carried out in patients with sepsis in the emergency department and find how the APACHE II score can be used for predicting ARDS in these patients.

Furthermore, we also found a strong relationship between the site of infection and the development of ARDS in patients with sepsis, including pancreatitis and pulmonary infection. In the case of pulmonary infection, ARDS may be caused by an indirect systemic inflammatory response of sepsis and direct lung injury. Individuals suffering from pancreatitis may experience ARDS as a result of a strong inflammatory response throughout the body, causing greater permeability of the epithelial and endothelial barriers and resulting in protein-rich exudates leaking into the alveolar space and interstitial tissues. This can affect oxygenation and gas exchange [22,23]. Therefore, we hypothesize that interventions need to implement more in patients with pulmonary infection and pancreatitis to improve the prognosis in these patients.

ARDS is a clinical syndrome that has a tendency to be frequently underdiagnosed, with nearly 40% of ARDS patients not being diagnosed quickly enough [24]. Improved comprehension of the risk factors of ARDS in patients with sepsis would enable clinicians to detect ARDS at an earlier stage. Additionally, various studies have altered the focus from clinical risk factors to biomarkers in order to predict the development of ARDS [25,26]. This is an approach towards more precise prediction and diagnosis for ARDS. CRP is one of the biomarkers assessed in the present meta-analysis. Two studies assessed this, and both studies have concluded that CRP is significantly higher in patients with ARDS. CRP is a protein produced by the liver in response to inflammation [27]. When the lungs are damaged, immune cells release cytokines and other inflammatory mediators, which can cause inflammation throughout the body. This inflammation triggers the liver to produce CRP, which can then be measured in the blood. It can be a useful marker of inflammation and can help clinicians monitor the severity of the inflammatory response in patients with ARDS [28].

The present meta-analysis has certain limitations. Firstly, only six studies were included in this meta-analysis. Among these six studies, only two were prospective cohort studies. The study was not developed to test the impact of a pre-specified exposure on a dependent variable to rather to systematically evaluated reported factors commonly measured in sepsis patients at the time of admission. Studies diagnosed sepsis using different sepsis scores, and due to limited studies available, we were not able to perform subgroup analysis.

## Conclusions

Based on the results of this systematic review and meta-analysis, it can be concluded that six factors are consistently and significantly associated with ARDS in patients with sepsis: SOFA score, APACHE II score, pulmonary sepsis, smoking, pancreatitis, and CRP. The pooled incidence of ARDS was 11.27%. Age, diabetes, and COPD were not found to be significantly associated with ARDS in this patient population. It is important for healthcare providers to consider these predictors when assessing patients with sepsis and septic shock to identify those at high risk for developing ARDS and implement appropriate preventive measures. Further studies are needed to confirm these findings and to explore additional potential predictors of ARDS in this patient population.
